# Ovarian Abscess; Operation, Recovery*Reported to the Louisville Clinical Society.

**Published:** 1897-07

**Authors:** August Schachner

**Affiliations:** Demonstrator of Anatomy in the Louisville Medical College; Visiting Surgeon to the Louisville City Hospital, etc., Louisville, Ky.


### Southern Medical Record.

A MONTHLY JOURNAL OF MEDICINE AND SURGERY.

VOL. XXVII. ATLANTA, GA., JULY, 1897. No. 7

### Original Articles.

### OVARIAN ABSCESS; OPERATION; RECOVERY.*

BY AUGUST SCHACHNER, M. D.

Demonstrator of Anatomy in the Louisville Medical College; Visiting Surgeon to the Louisville City Hospital, etc., Louisville, Ky.

I reported a case at the last meeting of this society about which there was some difference of opinion, especially as regards the evidence of iodoform poisoning. For the benefit of those who were not present at that time I will briefly detail the history.

The patient was a woman forty years of age, married, referred to me by Dr. Baker with the history of pelvic trouble which the doctors did not believe was the result of an abortion, although the patient claimed to have had such. Her husband had had gonorrhea two months before I saw her at the request of Dr. Baker, two weeks before the operation. At that time by vaginal examination I made out an enlarged uterus which was fixed on the right side. Her temperature at my first visit was slightly over 100° F. and between my first visit and the time of the operation two weeks later the temperature ranged from 99° and a fraction to 101° F. As already stated her husband had gonorrhea two months prior to the operation, having had frequent sexual intercourse with the woman during that time, and her trouble seemed to have dated from this gonorrheal infection. Diagnosis was made of small fibroid of the uterus, with some suppurative condition in the tubes or ovaries. Operation was advised and carried out at my infirmary. She was brought to the infirmary four

*Reported to the Louisville Clinical Society.

days before the operation, and at that time there was a slight vaginal discharge, although the vagina seemed fairly clean. Her temperature was then 100° F. and there was some tenderness in the right ovarian region. The vagina was washed thoroughly every day with sponges by the nurse, and her temperature taken each day. The first day there was only a slight vaginal discharge which increased each day going towards the operation. The morning of the operation she was given a bath, the vagina was thoroughly cleansed and packed with iodoform gauze. When put on the operating table at ten o'clock there was considerable discharge, of a thick muco-purulent character, in the vagina, having an offensive odor. This had occured in four hours. The vagina was again thoroughly washed with peroxide of hydrogen followed by bichloride of mercury solution, and the uterus also irrigated. The abdominal route was selected, and after making an incision I found a mass on the right side which proved to be the ovary and tube; there were some adhesions which were fairly dense, but there was no great difficulty in enucleation without rupture. The mass was ligated and removed, what served as the pedicle seemed to be nothing but thickened inflammatory tissue. On account of the woman's age, the tube and ovary on the opposite side, although they showed only slight evidence of disease, were also taken out. By some means a small vessel on the posterior surface of the uterus was ruptured, and there was slight oozing from this point. The cavity was irrigated before closing the abdomen with normal salt solution, and the parts were thoroughly examined preparatory to closing the wound. Pressure applied to the oozing point did not seem to control the hemorrhage; while the oozing was not great I thought advisable to introduce a strip of iodoform gauze. The piece I inserted was four to six inches wide and about twelve inches in length. It was twisted and packed not firmly but loosely behind the uterus. This gauze did not come in contact with the ligated portion of the ligament, which I believe was the impression by one speaker at the last meeting, as it was behind the uterus. After introducing this strip of gauze the abdomen was closed, and the patient left the table with a pulse of 100. The first eighteen hours after the operation the pulse gradually increased in frequency, reaching 156 to the minute, temperature 104° F. The woman had very little nausea, and mentally she was unusually clear. The rapid pulse, her settled state of mind, etc., was something that puzzled me at the time. The doctor who saw her with me believed it might be due to hemorrhage. In view of this fact we took off the dressing. I did not believe it was due to hemorrhage, but the dressing was removed and we withdrew the iodoform packing. We aspirated perhaps three drams of bloody fluid, and into the wound, reaching just down into the abdomen, a small strip of iodoform gauze was placed. This strip was one inch wide and three or four inches in length, just enough to act as a drain as far as the abdominal cavity. The wound was profusely dusted with iodoform, the dressing replaced, and over this bichloride gauze. The pulse remained over 120 to the minute for three days afterward, the temperature declining gradually, although she had some fever for several days. The woman went on without any further trouble. The stitches were removed on the seventh day; the wound had united perfectly, but later on, perhaps by early removal of the stitch, a small stitch abscess developed.

At the time I stated that I believed this condition was due to septic infection, mainly gonorrheal in its character, that the deeper structures had been invaded by the infection, and I take it that it was dependent upon conditions that were inherent at the time of the operation and not introduced from without. I take that view, first, because the husband had had an attack of gonorrhea two months before from the history given me; second, because of the peculiar condition of the uterine symptoms. On my first examination a considerable enlargement of the uterus was detected, but there was practically no uterine or vaginal secretion. As each day passed, the secretion increased in quantity until the last day it became muco-purulent in character, with considerable odor. I also believe that considering the rapidity with which the symptoms developed, it would be impossible for the condition to be due to any infection introduced from without, because the time was too short for the development of symptoms. I believe that the infection was latent within the uterus, and through manipulation or traumatism of the operation, a fresh infection occurred, the system becoming poisoned with the septic materials. Further in the discussion at the last meeting Doctors Wathen, Dugan and Samuels believed and strongly expressed themselves, that this could not be an infection, that it must be a case of iodoform poisoning. I do not believe it is a case of iodoform poisoning for several reasons. Dr. Dugan corrected me upon one point at the time as to the symptoms of iodoform poisoning which I accept, though I did not know that it ever existed in such a condition, and that is elevation of temperature. We know that in any intoxication, depending upon a poison taken from without, that the temperature is always rather depressed than elevated.

While we know that the peritoneum is one of the most absorbing membranes in the body, I believe unless this woman had an extreme idiosyncrasy in favor of iodoform, not enough of this agent was used to produce the symptoms from which she suffered. If she did have an idiosyncrasy in favor of iodoform, it is easily seen that it could not possibly be a case of iodoform poisoning, because before the symptoms disappeared, more iodoform was being used than while the alarming symptoms were manifest. If she were suffering from iodoform poisoning, the addition of more iodoform would hardly be expected to bring about an improvement. If a man were in coma from opium poisoning, you could not expect to improve his condition by giving him more opium at that time.

In discussing the case one speaker said the wall was protected by granulations; the iodoform was inserted too early for this to be true; granulations could not have formed to that degree to have protected the wall because the iodoform was placed in the cavity before the end of twenty-four hours. Therefore, there could not have been enough granulations to have protected the parts from further poisoning that might have occurred. At the time I reported the case I expressed my belief that the trouble was mostly gonorrheal in character, possibly a mixed infection, and I still believe that. This is only a belief, however, because it has not been verified by microscopical examination. Dr. Wathen, in discussing the case, said he did not think the gonococci invaded the deeper structures; this is my recollection of his statement. I think he is mistaken about this, as I have looked the matter up in Dennis' work, and several other authorities, and there is abundant evidence to show that the gonococci do invade the deeper structures, and may be carried to remote parts of the body.

DR. W. H. WATHEN: We all know that gonococci do enter the general system and are often found in the joints; but I made the statement at the previous meeting that the gonococci or their toxines would not go into the system and cause general infection by entrance as germs or by the absorption of the products of the growth of the gonococci. I still believe that there is not such a thing as a general septic infection, or a septic intoxication, from the entrance of gonococci from the uterus into the system through the lymphatics or veins, or by the absorption of the products of the growth of gonococci. In cases where the gonococci have been found in the joints, it is now claimed that when you have destroyed the specific germs in the local parts, that the gonococci disappear from the joints, indicating that the gonococci that invaded the system or that involved the joints were produced almost entirely from the local source and were taken into the system, and multiplied very little if at all in the structures about the joints. There is no reason why the local condition in the uterus should have taken on a sufficiently active septic condition to have caused such rapid septic infection as described. It is not true that the operation upon the pelvic structures caused a more rapid development of sepsis from the diseased uterus, but on the contrary probably retarded it, for the reason that large vessels and one or more chains of lymphatic vessels had been cut off by the ligatures that were applied. So that the septic matter, if in the system, must have been carried through the lymphatics and the veins at the base of the broad ligaments. I have never seen nor have I heard of such a development of sepsis following an operation of this kind, but if the fever, etc., were caused by sepsis I am inclined to think that this was possibly a case of septic intoxication, the result of the action of the germs of putrefaction upon the decidual structures following an abortion. To my mind the gonorrheal origin is absolutely impossible, and I do not believe even that it is possible to have such a form of sepsis in a woman who was suffering from septic effects following the operation. The explanation that it was caused by the presence of iodoform is a very rational one to me, because it developed just about the time we would expect iodoform poisoning to develop when we put the drug in contact with raw surfaces, and the symptoms are just as we would expect to find in a case of iodoform poisoning following a surgical operation. Dr. Schachner is mistaken in saying that granulations are not thrown out within the time mentioned; I might say not granulations but exudations; it is a well-known fact that within twenty-four hours we will often have decided exudations around a drainage tube introduced to the bottom of Douglas' pouch, or around gauze drains. This is a preventive of direct absorption, and anyone who has attempted to remove gauze from the peritoneal cavity within twenty-four hours, has found that the exudations were pretty firmly fixed, so that he could hardly accomplish removal. When the gauze was taken away, the condition of the cavity occupied by the gauze was so materially altered that doubtless not one-tenth as much iodoform could have been absorbed as would have been absorbed immediately.

